# Transglutaminase 2 regulates terminal erythroid differentiation via cross-linking activity

**DOI:** 10.3389/fcell.2023.1183176

**Published:** 2023-04-24

**Authors:** Yingying Zhang, Lifang Shi, Ke Yang, Xuehui Liu, Xiang Lv

**Affiliations:** ^1^ State Key Laboratory of Medical Molecular Biology, Haihe Laboratory of Cell Ecosystem, Department of Pathophysiology, Institute of Basic Medical Sciences, Chinese Academy of Medical Sciences & Peking Union Medical College, Beijing, China; ^2^ Changping Center for Disease Control and Prevention, Beijing, China

**Keywords:** TGM2, erythroid differentiation, fetal liver, cross-linking activity, cell cycle

## Abstract

Transglutaminase 2 (TGM2) is a versatile enzyme that modulates cell survival and differentiation. However, its role in terminal erythroid differentiation is poorly understood. In this study, we investigated the function of TGM2 in primary fetal liver erythroid differentiation. We predicted TGM2 as an upstream regulator via ingenuity pathway analysis (IPA), and found that its expression was increased at both RNA and protein level during terminal erythroid differentiation. TGM2 cross-linking activity inhibitors GK921 and Z-DON suppressed erythroid maturation and enucleation, while its GTPase inhibitor LDN27219 had no such effect. Z-DON treatment arrested differentiation at basophilic erythroblast stage, and interfered with cell cycle progression. RT-PCR demonstrated decreased GATA-1 and KLF1, and disarranged cyclin, CDKI and E2F family genes expression after Z-DON treatment. In conclusion, TGM2 regulates terminal erythroid differentiation through its cross-linking enzyme activity.

## 1 Introduction

Mature red blood cells are produced from hematopoietic stem cells (HSCs), which commit to erythroid progenitors followed by terminal erythroid differentiation. It begins with proerythroblasts (Pro), which sequentially divide into basophilic erythroblasts (Baso), polychromatic erythroblasts (Poly) and orthochromatic erythroblasts (Ortho) that enucleate to generate reticulocytes following cell cycle exit. Finally, reticulocytes mature into erythrocytes by removing intracellular organelles and reorganizing cellular membrane. During terminal erythroid differentiation, erythroblasts undergo several notable changes, including a decrease in size, nuclear and chromatin condensation, and hemoglobinization, which are precisely regulated at multiple levels (Hattangadi et al., 2011; Wong et al., 2011). Erythroid-specific transcriptional factors such as GATA1 (Gutiérrez et al., 2020), KLF1/EKLF (Caria et al., 2022), TAL1 (Hoang et al., 2016), and NFE2 (Gasiorek et al., 2015) are involved in this process. Although multiple regulators and signaling pathways have been revealed to participate terminal erythroid differentiation (Mei et al., 2021; Yijie Liu et al., 2021), and the stepwise changes in chromatin accessibility (Schulz et al., 2019) as well as in transcriptomics (An et al., 2014; Ludwig et al., 2019)/ proteomics (Gautier et al., 2016; 2020) have been elucidated, the mechanisms that drive successful maturation and enucleation remain largely undefined.

Transglutaminase 2 (TGM2) is the most ubiquitously expressed member of the transglutaminase family proteins. It is composed of an NH2-terminal *β*-sandwich domain, a catalytic core domain and two COOH-terminal *β*-barrels, and is localized both intracellularly and extracellularly (Lorand et al., 2003). TGM2 modulates biological events such as cell survival, differentiation and tumor formation, via multiple enzymatic activities including cross-linking, guanosine 5′-triphosphate (GTP) hydrolysis, scaffolding, protein disulfide isomerization, serotonylation (Farrelly et al., 2019) and protein kinase activity (Greenberg et al., 1991; Nakaoka et al., 1994; Eckert et al., 2014). Notably, Ca^2+^-dependent protein cross-linking activity is one of the main functions of TGM2 and can be negatively regulated by GTP (Lee et al., 2017). TGM2 is a promising drug target considering its important roles in cancer. A variety of inhibitors specific to distinct TGM2 activities have been developed. For example, GK921 (Ku et al., 2014) and Z-DON(Schaertl et al., 2010) inhibit TGM2 cross-linking enzyme activity. LDN27219 (Case et al., 2007) suppresses GTPase activity. ZED-1227 (Schuppan et al., 2021) inhibits TGM2 expression and activation under inflammatory conditions.

TGM2 knockout mice exhibit mild anemia with reduced RBC counts and hematocrit, indicating that TGM2 may be involved in erythropoiesis (Bernassola et al., 2002). Several *in vitro* studies using human chronic myelogenous leukemia K562 cells have shown that TGM2 mediates erythroid differentiation by translocation to cell membrane. TGM2 is recruited to cell membrane by Glycosphingolipid GD3 in response to erythroid inducer hemin and all trans-retinoic acid (tRA), and its overexpression promotes hemoglobin accumulation (Kang et al., 2004). The α1-adrenergic receptor (α1-AR)/TGM2 signaling pathway-directed GD3 production is a crucial step in erythroid differentiation of K562 cells (Ha et al., 2017). However, the function and mechanism of TGM2 in terminal differentiation of primary erythroid progenitors remain to be demonstrated.

In this study, we identified that TGM2 is a potential upstream regulator of terminal erythroid differentiation by analyzing the transcriptome data of both human and mouse erythroblasts. Using fetal liver derived *in vitro* terminal erythroid differentiation system and three different types of TGM2 inhibitors, we demonstrated that inhibition to the cross-linking enzyme activity of TGM2 specifically arrested terminal erythroid differentiation at the Baso stage. Cell cycle and transcriptional analysis implied impaired cell cycle progression upon the inhibition.

## 2 Materials and methods

### 2.1 Reagents and antibodies

Three inhibitors were used in this study. LDN27291 was purchased from TOCRIS. GK921 was from MedChemExpress and Z-DON was from Zedira. The inhibitors were added respectively to *in vitro* cultured mouse fetal liver derived erythroid cells at the beginning (D0) and day 2 of differentiation (D2).

The following antibodies were from BD Bioscience: CD16/CD32 (mouse BD Fc Block), FITC Rat Anti-mouse Ter119, PE Rat Anti-mouse CD44, APC-Cy7 Rat Anti-mouse GR1, APC-Cy7 Rat Anti-mouse CD45, APC-Cy7 Rat Anti-mouse CD11b and APC Rat Anti-mouse CD71. Rabbit Anti-mouse TGM2 and PE Anti-mouse TGM2 were from Abcam and Novusbio respectively. FITC Annexin V Apoptosis Detection Kit were from Biolegend.

### 2.2 Mice

The mouse experiments were approved by the Animal Ethics Committee of the Institute of Basic Medical Sciences, Chinese Academy of Medical Sciences & Peking Union Medical College (No. ACU​C-A01-2021-032). Male C57BL/6 mice aged 8 weeks maintained under specific pathogen-free conditions were used in this study.

### 2.3 Fluorescence-activated cell sorting of bone marrow erythroblasts at distinct stages

Bone marrow cells were isolated from 8-week C57/BL6 male mice and filtered with 70-μm Nylon membrane to obtain single-cell suspensions in modified phosphate buffered saline (PBS/2 mM EDTA/0.5% BSA). CD45-cells were purified with anti-CD45 MicroBeads (Miltenyi Biotec) through LS Columns (Miltenyi Biotec) according to the manufacturer’s instructions. The CD45-cells were incubated with CD16/CD32 for 15 min on ice. Samples were subsequently stained with Ter119-FITC (0.5 μg/106 cells), CD44-APC (0.2 μg/106 cells), CD45-APC-Cy7 (0.1 μg/106 cells), CD11b-APC-Cy7 (0.1 μg/106 cells), and GR1-APC-Cy7 (0.1 μg/106 cells) on ice for 20 to 30 min in the dark. Finally, cells were stained with the viability marker 7-AAD (0.25 μg/106 cells) on ice for 10 min in the dark. Cells were sorted using BD FACS Aria SORP flow cytometry. CD44hiTer119^low^ population are proerythroblasts, and plot of CD44 versus FSC of the Ter119 positive cells revealed 5 naturally occurring cell cluster, which were gated as basophilic erythroblasts, polychromatic erythroblasts, orthochromatic erythroblasts, reticulocytes, and mature red cells, respectively (Jing Liu et al., 2013).

### 2.4 RNA sequencing and data analysis

About 10^5^-10^6^ cells were collected and centrifuged at 500 g at 4°C for 10 min. The supernatant was discarded and cells were lysed by Geek Cell lysates A&B. cDNA libraries were prepared using KAPA Hyper Prep Kits (Roche, KK8504) based on Illumina platform, sequenced by PE150 based on Illumina NovaSeq platform. The transcriptome data (GSE229589) of terminal erythroid differentiation of mouse bone marrow were produced by our laboratory. Transcriptome data of human fetal liver or peripheral blood CD34^+^ cells derived erythroblasts were from published work (GSE102182) (Huang et al., 2017). Quality of data was controlled by FastQC, adaptor sequence and low-quality sequence were removed by Cutadapt and Trimmatic respectively, data were then compared to Mouse mm10 genome by Hisat2. Comparison results were gathered and counted by Samtools and FeatureCounts before matrix of gene expression was constructed. Differentially expressed genes were analyzed by DESeq2. The upstream regulatory factors of differentially expressed genes were predicted using ingenuity pathway analysis (IPA). Z-score >2 or < −2 is considered significant.

### 2.5 *In vitro* terminal erythroid differentiation of mouse fetal liver Ter119^-^ cells

Fetal livers were isolated from embryos at day 14.5 from wild-type C57/BL6 pregnant mice. Ter119^-^ cells were purified with anti-Ter119 MicroBeads (Miltenyi Biotec) through LD Columns (Miltenyi Biotec) according to the manufacturer’s instructions. Ter119^-^ cells were cultured in FL proliferation medium (Iscove’s modified Dulbecco’s medium (IMDM) containing 1% 3K, 25% FBS, 1% BSA, 2 mM L-glutamine, 10 μg/mL recombinant human insulin, 2 mM L-glutamine, and 10^–4^ M *β*-mercaptoethanol, 10^–6^ M Dexamethasone, 20 ng/mL IL-3, 0.4% Cholesterol, 40 ng/mL IGF-1, 200 μg/mL holotransferrin, 10 U/mL Epo and 100 ng/mL SCF) for 3 days. Subsequently the medium was replaced with FL differentiation medium (IMDM containing 1% 3K, 5% FBS, 3 U/mL Epo, 100 ng/mL SCF, 500 μg/mL holo-transferrin, 0.00127% 1-Thioglycerol, 10% Serum Replacement and 10% PFHM-II) to induce terminal erythroid differentiation for 4 days.

### 2.6 Western blotting analysis

Pellets of *in vitro* erythroid differentiated mouse fetal liver Ter119^-^ cells were collected and lysed in RIPA buffer with PMSF and cocktail. Following incubation on ice with occasional rotation, the lysate was centrifuged at 12,000 rpm at 4°C for 15 min, and the protein concentration in the supernatant was quantified using a Pierce BCA protein assay kit (Tanon). Approximately 10–30 μg of total extract proteins were loaded into each lane of a 12% SDS-PAGE gel. The PVDF membranes were incubated overnight with the primary antibodies and then probed with the corresponding secondary antibodies for 1–2 h at room temperature. The protein bands were visualized using an ECL chemiluminescence kit.

### 2.7 RNA isolation and RT-qPCR

Total RNA was extracted from mouse fetal liver derived erythroid cells treated with different concentrations of inhibitors using TRIzol Reagent. First strand cDNA was synthesized using 1 μg of RNA as a template, M-MLV reverse transcriptase, and random primers. Real-time RT-PCR amplifications were performed using an iQTM5 Real-Time PCR Detection System (Bio-Rad), with SYBR Green PCR Mix, and the forward/reverse primer pairs were shown in Supplementary Table S1. The qPCR amplification conditions included 95 °C for 10 min, followed by 39 cycles of amplification at 95°C for 10 s, 60°C for 30 s, 72°C for 30 s, and 72°C for 5 min as the final elongation step. Gene expression was quantified by normalizing the data against the GAPDH control values using the delta Ct method.

### 2.8 Flow cytometry analysis

One million cells were incubated with 0.8 μL anti-mouse Ter119-FITC and 0.6 μL anti-mouse CD71-APC antibodies for 30 min at 4°C, followed by incubation of Hoechst33342 (Beyotime) or Reddot1 (Biotium) for 10 min at 4°C to assess the efficiency of erythroid differentiation and enucleation by flow cytometry on LSR-Fortessa (Zhang et al., 2003; Han et al., 2022).

### 2.9 TGM2 cross-linking enzyme activity assay

TGM2 cross-linking enzyme activity was detected using TGM2 enzyme activity detection kit (Novus) according to the manufacturer’s instruction.

### 2.10 Cell cycle analysis

Cell cycle analysis was conducted using cell cycle detection kit (KeyGEN BioTECH). Briefly, one million cells were fixed with cold ethanol overnight, followed by incubation with PI/RNase A for 30 min at room temperature to be detected on a BD Accuri C6 and analyzed by ModFit LT.

### 2.11 May-Grunwald Giemsa staining and analysis

Cells were collected and spun onto glass slides using cytospin, and stained by May-Grunwald (Sigma) solution for 5 min, and immediately transferred to 10% Giemsa solution (freshly prepared) for 10 min, finally rinsed gently with deionized water twice, dried, sealed with gum. The slides were examined and photographs were taken under an optical microscope. The pictures were analyzed by Image-Pro, and the cells at different differentiation stages were distinguished according to the cell size.

### 2.12 Statistical analysis

Data are expressed as the mean ± standard deviation. Statistical analysis was performed using unpaired Student’s t-test for comparison between two groups. Multiple-group comparisons were analyzed by one-way ANOVA in Prism 9. *p* values of less than 0.05 were considered statistically significant.

## 3 Results

### 3.1 TGM2 is a potential upstream regulator of terminal erythroid differentiation

**FIGURE 1:**
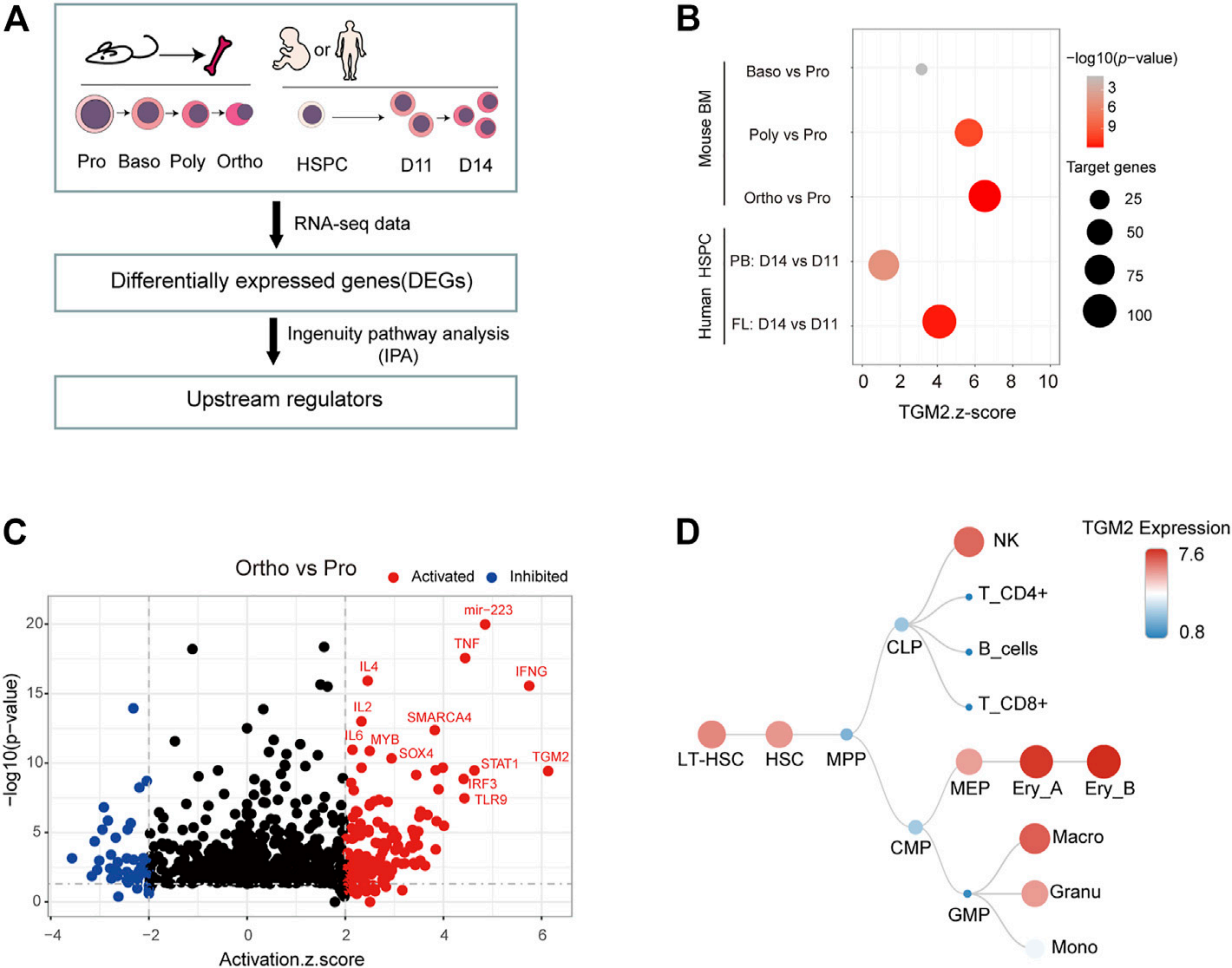
TGM2 is activated and highly expressed in terminal erythroid differentiation. **(A)** Flow chart for predicting upstream regulators of terminal erythroid differentiation by IPA analysis. Human Data were from GSE102182; Mouse data were from DOI:10.12213/11.A001L.202105.366.V1.0. **(B)** Dot plot showing the Z-score and *p*-value of TGM2 as the upstream regulator from IPA analysis of terminal erythroid differentiation. **(C)** Upstream regulators predicted by IPA analysis using ‘Ortho vs. Pro’ differential genes. **(D)** TGM2 is highly expressed in erythroblasts among the hematopoietic hierarchy according to data from normal mouse hematopoietic cells (GSE60101).

We isolated mouse bone marrow cells and sorted erythroblasts of different maturation stages using flow cytometry (Jing Liu et al., 2013). May-Grunwald Giemsa (MGG) staining showed the representative images of Pro, Baso, Poly and Ortho cells, respectively (Supplementary Figure S1). Transcriptomic analysis of these four stages of cells was performed. To predict upstream regulators of terminal erythroid differentiation, differentially expressed genes (DEGs) from “Baso vs. Pro”, “Poly vs. Pro”, “Ortho vs. Pro” were subjected to ingenuity pathway analysis (IPA) respectively (Figure 1A). IPA core analysis revealed that TGM2 was activated in all the three pairwise comparisons, and the Z-score increased along with the differentiation process (Figure 1B). Notably, TGM2 ranked first in the upstream regulators predicted from the “Ortho vs. Pro” group (Figure 1C, Supplementary Table S2). Additionally, using transcriptome data of *in vitro* erythroid differentiated human CD34^+^ cells, either from fetal liver or from peripheral blood (GSE102182), TGM2 was predicted as an upstream regulator for both “fetal_d14 vs. fetal_d11”, and “adult_d14 vs. adult_d11” groups (Figures 1A, B).

**FIGURE 2:**
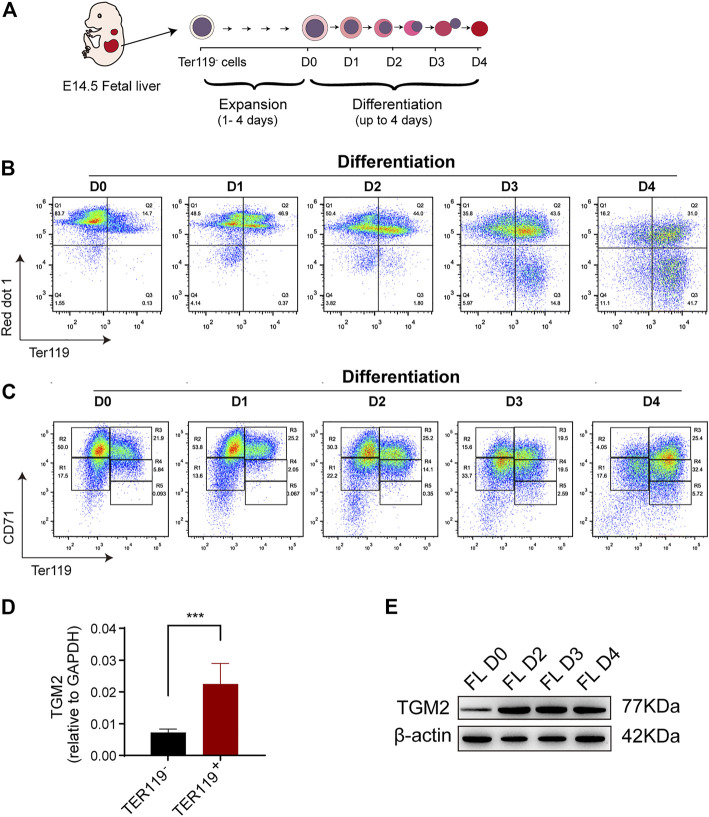
TGM2 expression is elevated in mouse fetal liver erythroid differentiation system. **(A)** Schematics indicated the process of Ter119^-^ cells isolation, *in vitro* expansion and erythroid differentiation from mouse fetal liver. **(B, C)** Flow cytometry analysis of *in vitro* fetal liver erythroid differentiation. **(B)** Enucleation efficiency were evaluated using TER119/red dot1 staining patterns. **(C)** Cells were sorted into 5 separate populations corresponding to progressive stages of definitive erythroid development using CD71/TER119 staining patterns (regions R1-R5). There are mainly primitive progenitor cells (including mature BFU-Es and CFU-Es) in R1, Pro and early Baso in R2, early and late Baso in R3, Poly and early Ortho in R4, and late Ortho and reticulocytes in R5. **(D)** RT-PCR showing significantly higher TGM2 expression in TER119^+^ cells than that in TER119^-^ cells isolated from E14.5 fetal liver (n = 3). Data indicate means with bar as SD. ^∗∗∗^, *p* < 0.001, *t*-test. **(E)** Western blotting showing increased TGM2 protein after terminal erythroid differentiation of *in vitro* cultured fetal liver cells. (FL, fetal liver; D0,2,3,4 means Differentiation for 0,2,3,4 days, respectively).

TGM2 was shown highly expressed in erythroblasts according to dataset GSE60101 from mouse normal hematopoietic cells (Figure 1D). Using *in vitro* fetal liver erythroid differentiation system (Figures 2A–C), we showed that mRNA expression of TGM2 in fetal liver derived Ter119^+^ cells was significantly higher than that in Ter119^-^ cells (Figure 2D). In addition, protein expression of TGM2 increased significantly at day 2 after erythroid differentiation, and maintained in a high level thereafter (Figure 2E). Considering the results of IPA analysis and that TGM2 expression was elevated atterminal erythroid differentiation, it is suggested that TGM2 might be an important upstream regulator in terminal erythroid differentiation.

### 3.2 Inhibition of TGM2 cross-linking activity impairs terminal erythroid differentiation and enucleation

TGM2 mainly functions via its GTPase and cross-linking enzyme activity. To explore the role of TGM2 in erythroid differentiation, three inhibitors were used, as indicated in Supplementary Figure S2. Specifically, LDN27219 is a reversible slow-binding TGM2 inhibitor that binds to the GTP site of the enzyme and inhibits its GTPase activity (Case et al., 2007). GK921 is a reversible TGM2 cross-linking inhibitor (Ku et al., 2014), and Z-DON is an irreversible TGM2 cross-linking inhibitor (Schaertl et al., 2010). The inhibitors were added twice at the beginning (D0) and day 2 (D2) of differentiation, erythroid differentiation and enucleation efficiency was detected at Day 4 (D4).

**FIGURE 3:**
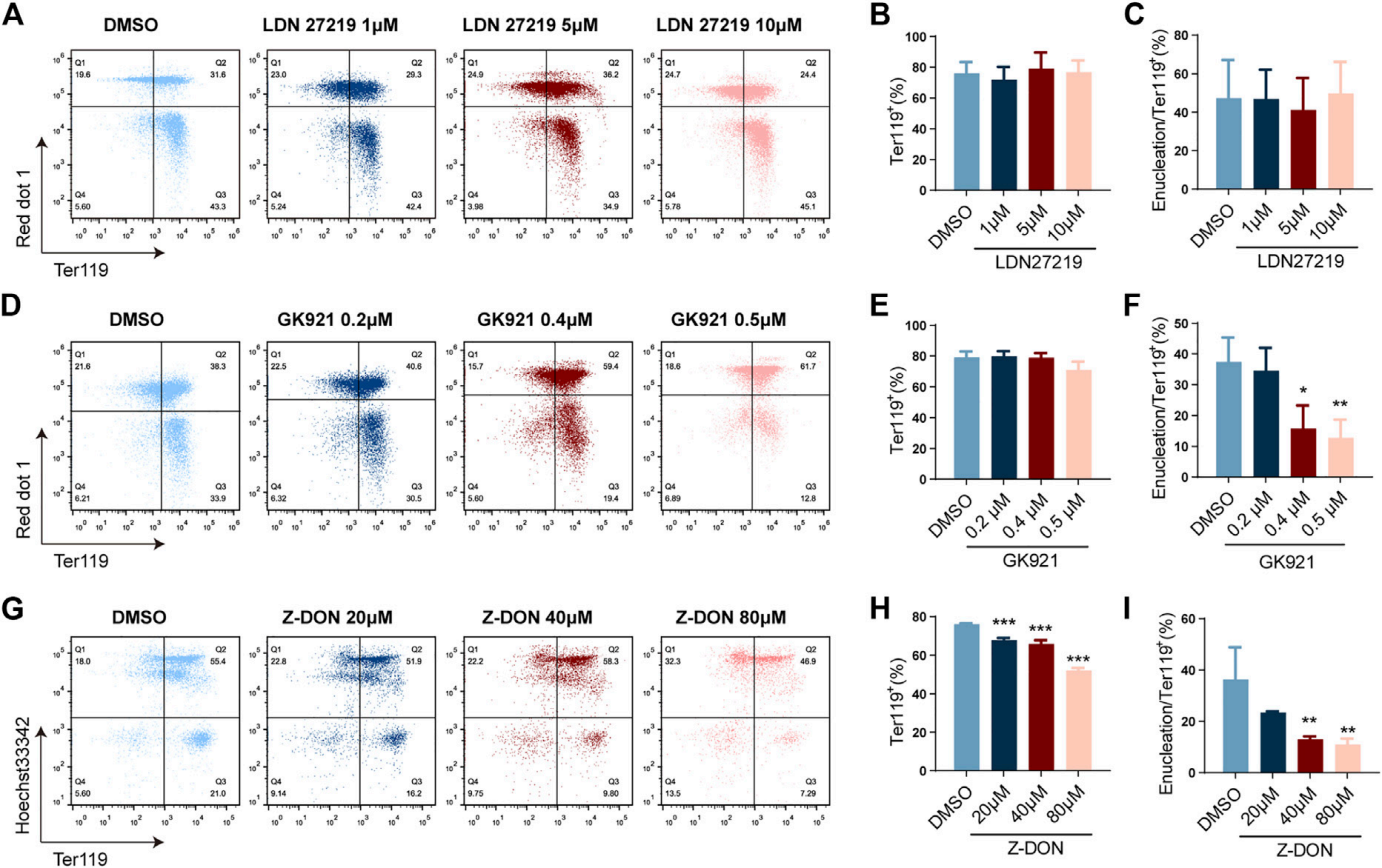
Inhibition of TGM2 cross-linking activity suppresses terminal erythroid differentiation and enucleation. **(A, D, G)** Flow cytometry analysis showing the effects of TGM2 inhibitors LDN27219**(A)**, GK921**(D)** and Z-DON**(G)** on terminal erythroid differentiation and enucleation of *in vitro* differentiated mouse fetal liver cells. **(B, C, E, F, G, I)** Quantification of Ter119^+^ cells and enucleation ratio in LDN27219 **(B, C)**, GK921 **(E, F)** and Z-DON **(G, I)** treatment groups (n = 3 each). Data indicate means with bar as SD. ^∗^, *p* < 0.05, ^∗∗^, *p* < 0.01, ^∗∗∗^, *p* < 0.001, One-way ANOVA.

LDN27219-treated group showed no significant change in TER119^+^ cell proportion and enucleation ratio compared to control group at all three concentrations tested (Figure3A-C), suggesting that the GTPase activity of TGM2 is dispensable for erythroid differentiation. In contrast, inhibitors targeting TGM2 cross-linking activity suppressed both erythroid differentiation and enucleation of mouse fetal liver cells. Specifically, for the GK921-treated group, while proportion of TER119^+^ cells did not change significantly, enucleation ratio markedly decreased in the high drug concentration group (0.4 μM and 0.5 μM) (Figures 3D-F). Of note, Z-DON, the irreversible TGM2 cross-linking inhibitor, dose-dependently suppressed both terminal erythroid differentiation and enucleation of the cells (Figures 3G-I). These data together implied that the crosslinking activity of TGM2 is important for terminal erythroid differentiation and enucleation.

### 3.3 Inhibition of TGM2 cross-linking activity arrests terminal erythroid differentiation at the Baso stage

**FIGURE 4:**
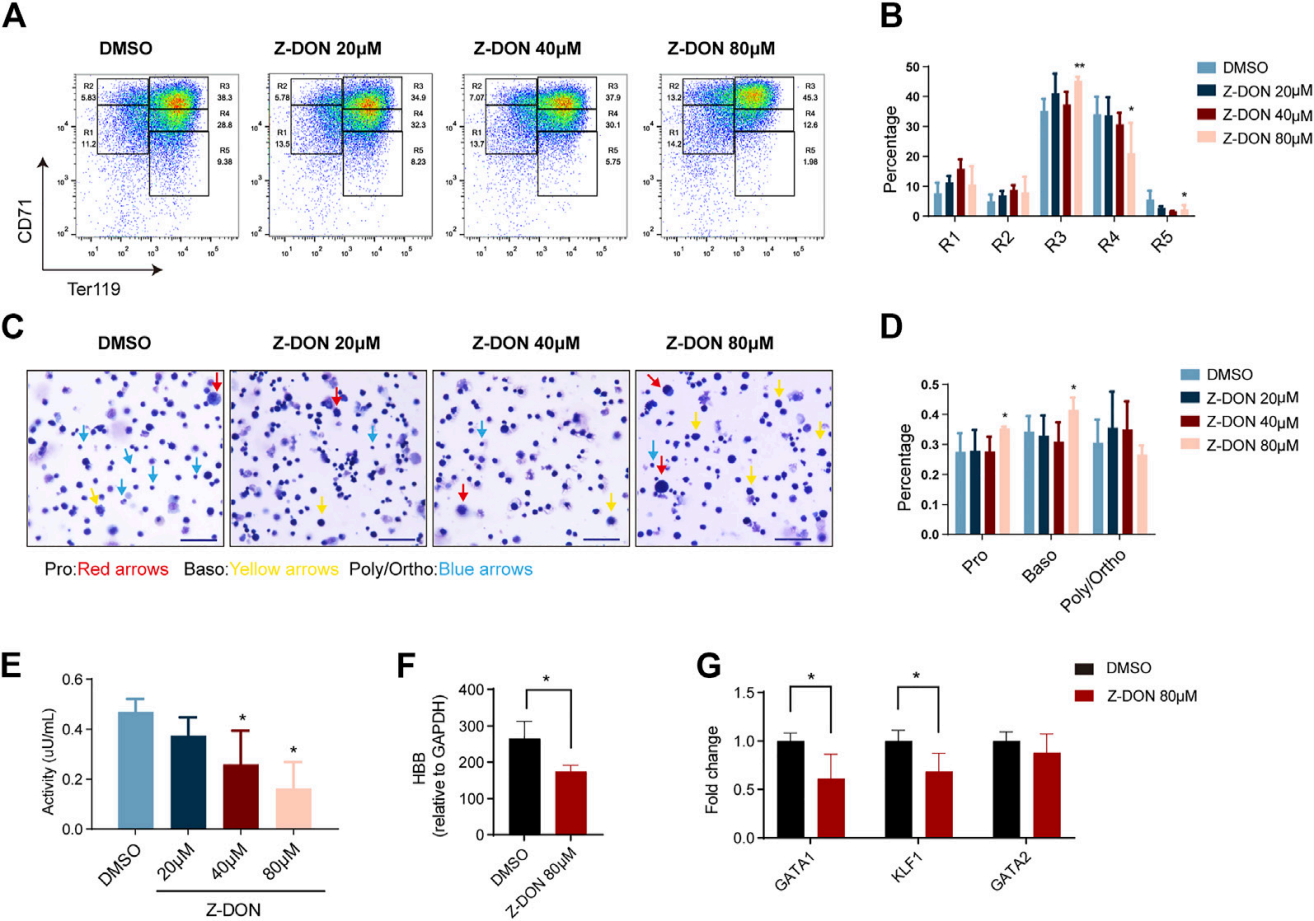
Inhibition of TGM2 cross-linking activity arrests terminal erythroid differentiation at the Baso stage. **(A, B)** Flow cytometry analysis and quantification of compartments R1-R5 via using CD71/TER119 staining patterns from in the DMSO and Z-DON group (n = 7). Data indicate means with bar as SD. ^∗^, *p* < 0.05, ^∗∗^, *p* < 0.01, One-way ANOVA. **(C)** May-Grunwald Giemsa (MGG) staining of cells differentiated for 4 days in the DMSO and Z-DON group. Photos were taken with a ×20 objective of Zeiss brightfield microscope. Scale bar, 50 µm. Pro, Baso, Poly/Ortho cells are indicated with red, yellow and blue arrows, respectively. **(D)** Quantification of Pro, Baso, Poly/Ortho in the DMSO and Z-DON group. Photos from 4-7 views with 150 cells per field of view were quantified. Data indicate means with bar as SD. ^∗^, *p* < 0.05, One-way ANOVA. Cell diameter range is as follows: Pro, 11–13μm; Baso, 9–11μm; Poly-Ortho, 7–9 μm. **(E)** TGM2 cross-linking enzyme activity was detected before and after Z-DON treatment. Data indicate means with bar as SD. ^∗^, *p* < 0.05, One-way ANOVA. **(F–G)** RT-PCR showing the expression of HBB **(F)** and key transcriptional factors **(G)** in the 80 μM Z-DON and control groups. Data indicate means with bar as SD. ^∗^, *p* < 0.05, *t*-test.

To evaluate the effect of Z-DON on terminal erythroid differentiation in more details, cells were double stained for TER119 and CD71 protein expression, which divided the whole population into five consecutive differentiation stages of R1 to R5. Among them R1 mainly represents progenitor cells including BFU-Es and CFU-Es, R2 represents Pro and early Baso, R3 for early and late Baso, R4 for Poly and early Ortho, and R5 for late Ortho and reticulocytes (Zhang et al., 2003). We found that the ratio of R3 was significantly increased, and that of R4 and R5 were decreased in the 80 μM Z-DON-treated group, suggesting arrested terminal erythroid differentiation at the Baso stage (Figure4A, B). Increases in R1 and R2 were also observed, implying that Z-DON also affected earlier erythroid differentiation. MGG staining further supported that the proportion of early-stage erythroblasts increased significantly in the 80 μM Z-DON-treated group (Figures 4C, D and Supplementary Figure S3A). Cell apoptosis was examined and assay was performed and we found that cell apoptosis was showed not increased after Z-DON treatment (Supplementary Figure S3B).

Total cell protein extract from Z-DON-treated cells showed a dose-dependent decline in cross-linking enzyme activity as expected. The highest dose of 80 μM Z-DON treatment led to a ∼60% of inhibition (Figure 4E). We further examined expression changes of erythroid genes and transcriptional regulators upon the inhibition. 80 μM Z-DON treatment suppressed mRNA expression of *β*-globin gene by 30%–40% (Figure 4F). The key erythroid transcriptional factors GATA1 and EKLF/KLF1, which are highly expressed at Baso stage (Supplementary Table S3), were significantly downregulated. Whereas the expression of hematopoietic stem/progenitor cell regulator GATA2 was roughly unchanged (Figure 4G).

### 3.4 Inhibition of TGM2 cross-linking activity leads to dysregulated cell cycle progression in terminally differentiated erythroid cells

**FIGURE 5:**
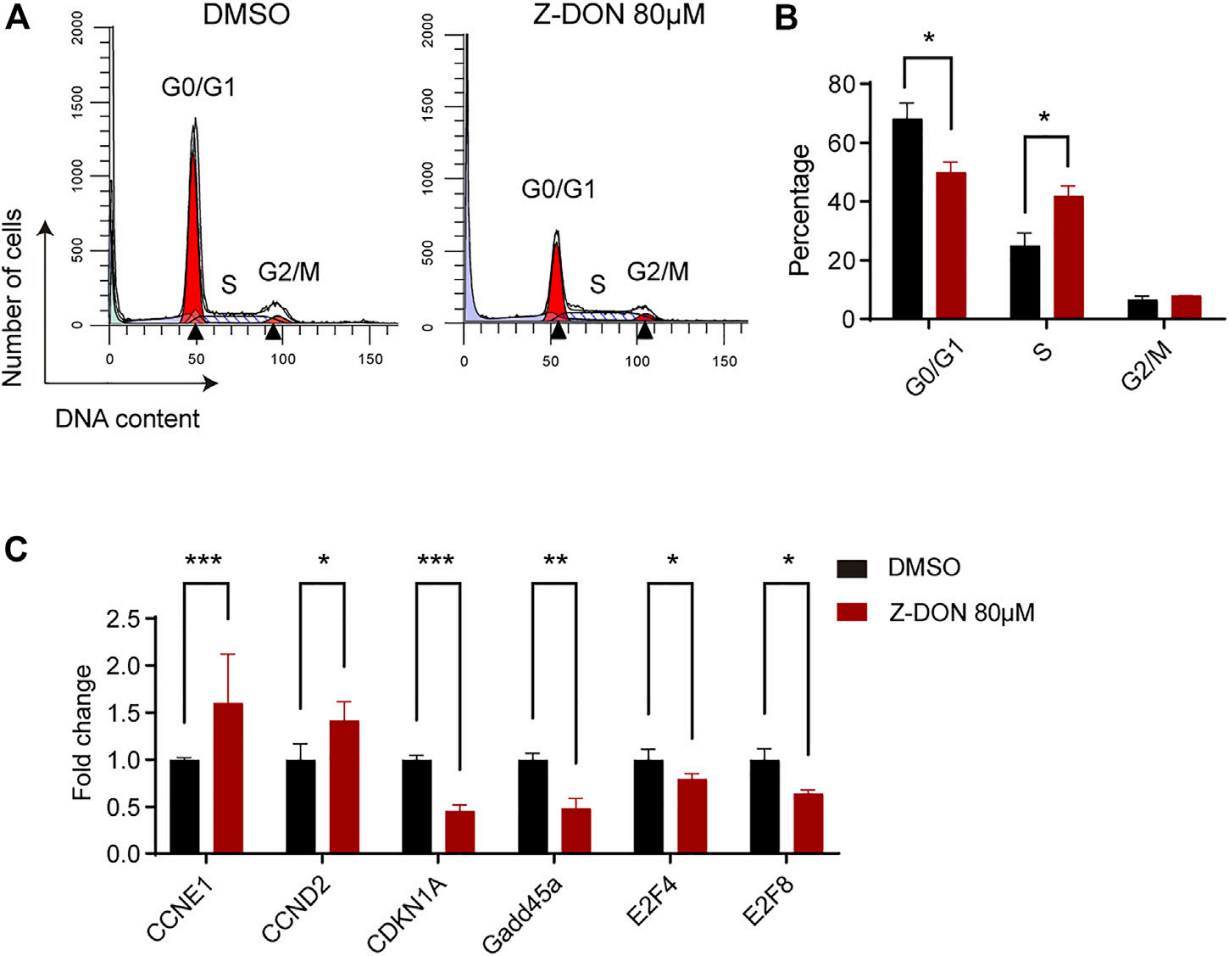
Inhibition of TGM2 cross-linking activity leads to dysregulated cell cycle progression. **(A, B)** Flow cytometry analysis **(A)** and quantification **(B)** of cells in G0/G1, S and G2/M phases in the DMSO and Z-DON group (n = 3). Black triangles in **(A)** mark mean DNA content of cells in G0/G1 and G2/M phase. Data indicate means with bar as SD. ^∗^, *p* < 0.05, *t*-test. **(C)** RT-PCR showing expression levels of indicated cell cycle related genes in the DMSO and Z-DON group (n = 4). The GAPDH was used as an internal reference gene. Result was shown as fold change normalized to DMSO group. Data indicate means with bar as SD. ^∗^, *p* < 0.05, ^∗∗^, *p* < 0.01, ^∗∗∗^, *p* < 0.001, *t*-test.

Flow cytometry analysis showed that the percentage of G0/G1 phase cells decreased (50.1% ± 2.7% vs 66% ± 4.2%), and the percentage of cells in S phase increased (41.9% ± 2.7% vs 25% ± 3.3%) in the 80 μM Z-DON-treated group (Figures 5A, B). Particularly, we noticed a slight increase in DNA content of G0/G1-, late S- and G2/M-phase cells in the Z-DON group. This together suggested dysregulated cell cycle progression. Transcriptional analysis of genes involved in G1 to S progression revealed that Cdkn1a [inhibitor of G1/S phase transition (Taniguchi et al., 1999)] and Gadd45a [genotoxic stress response, cell cycle arrest, and cell differentiation (Wingert et al., 2016)] expression decreased, while Ccne1 [regulator of CDK2, G1/S phase transition gene (Geng et al., 2003; Berthet et al., 2006; Sen et al., 2021)] and Ccnd2 [cell cycle regulator (Halsey et al., 2012)] upregulated in the 80 μM Z-DON-treated group, consistent to the changes in cell cycle proportion and the arrest of cells at Baso stage (Figure4C, Supplementary Table S3). E2F proteins are major transcriptional regulators required to coordinate cell cycle progression (Kinross et al., 2006). The repressive E2F4 and E2F8 facilitate the G0/G1 cell cycle exit via transcriptional repression of genes that promote S-phase progression (Li et al., 2008; Miller et al., 2010), and are highly expressed in Baso stage erythroblasts (Supplementary Table S3). We showed here that the expression of E2F4 and E2F8 genes was also decreased after Z-DON treatment (Figure 5C).

## 4 Discussion

Our study uncovered that TGM2 plays a key role in terminal erythroid differentiation through its cross-linking activity. We predicted TGM2 as a potential upstream regulator in terminal erythroid differentiation based on transcriptional data from mouse bone marrow erythroblasts, and human primary erythroid cells. Consistently, TGM2 is highly expressed in terminal erythroid differentiation. Among the multiple enzymatic activities of TGM2, we found that targeting cross-linking function specifically affects terminal differentiation and enucleation of mouse fetal liver derived erythroid cells, especially by the irreversible inhibitor Z-DON. The cells were arrested at Baso stage upon Z-DON inhibition, accompanied by dysregulated erythroid genes expression and cell cycle progression. The study implied TGM2 and its cross-linking activity as the potential targets for efficient terminal erythroid differentiation *in vitro* and for etiology research of anemic diseases.

Early studies using K562 cells established a link between TGM2 and erythroid differentiation (Kang et al., 2004; Ha et al., 2017). However, K562 (Klein et al., 1976), as well as HUDEP2 (Kurita et al., 2013)and MEL (Scher et al., 1977) models may not be appropriate for studies on terminal erythroid differentiation, as their differentiation is arrested at early erythroblast stage, and show low efficiency of enucleation. Therefore, we employed the terminal erythroid differentiation system derived from mouse fetal liver, which reached 40% enucleation rate. TGM2 expression was significantly elevated in this system, suggesting it is a suitable model to study the function of TGM2 during terminal erythroid differentiation. Using the system, we showed that TGM2 played an important role in promoting terminal erythroid differentiation, consistent with the results from K562 (Kang et al., 2004). Moreover, inhibition of TGM2 activity mainly blocked the differentiation from Baso to Poly and Ortho, and impaired erythroid enucleation. A weaker effect to earlier erythroid differentiation was also observed, implying multiple functions of Z-DON at different stages of erythroid differentiation. Additionally, TGM2 is also expressed at MEP stage (Figure 1D), further investigation will be needed for a more comprehensive understanding to the erythroid function of TGM2.

Both cross-linking enzyme inhibitors Z-DON and GK921 significantly suppressed terminal erythroid differentiation, but GTPase inhibitor LDN27219 (Schuppan et al., 2021) had no effect, suggesting the necessity of TGM2 cross-linking activity for the process. Consistently, effect of the irreversible inhibitor Z-DON was stronger than that of GK921, which binds to TGM2 reversibly (Katt et al., 2018). The cross-linking activity of TGM2 leads to intra/inter-molecular isopeptide bond, deamidation, as well as multiple types of protein modifications, which are found important for increasingly more pathophysiologic events (Farrelly et al., 2019; Wang et al., 2022; Zheng et al., 2023). We showed that the expression of HBB, as well as the central erythroid transcriptional regulators GATA1 and KLF1 were downregulated in the Z-DON group. Caspase-mediated degradation of GATA1 also arrested erythroid differentiation at the Baso stage (Lefèvre et al., 2017). KLF1 is critical for late stage maturation and enucleation of erythroblasts (Gnanapragasam et al., 2016). TGM2 cross-links caspase 3, transamidates and protects retinoblastoma tumor suppressor protein (Rb) (Yamaguchi et al., 2006; Mishra et al., 2007), another regulator of terminal erythroid differentiation. The exact substrates of TGM2 cross-linking activity and the downstream signaling pathways in terminal erythroid differentiation still pending identification.

We showed here that Z-DON treatment resulted in disturbed cell cycle progression. The decreased G0/G1-to S-phase proportion, downregulated Cdkn1a, Gadd45a, plus upregulated Ccne1 and Ccnd2 genes likely suggested enhanced G1/S transition with Z-DON treatment, or may represent a consequence of differentiation arrest at Baso stage. E2F family factors E2F4 and E2F8 expression are normally more enriched in Baso than in later stages, their downregulation in the Z-DON group may therefore be upstream to the differentiation arrest. E2F4 is the major repressor of E2F family. While majority studies focused on the cell cycle exit function of E2F4, it was shown to promote cell proliferation in fetal liver erythropoiesis and in mouse embryonic stem cells (Kinross et al., 2006; Hsu et al., 2019). Particularly, the Baso stage arrest of Z-DON-treated cells observed here phenocopies the macrocytic anemia associated with E2F4 knockout mice (Kinross et al., 2006). The atypical repressor E2F8 is also involved in S-G2/M transition and polyploidization formation (Pandit et al., 2012). Atypical E2Fe in plant was reported to protect mitotic cyclins from degradation (Lammens et al., 2008). Those together with the observation of elongated late S-phase and increased DNA content in both G2/M and G0/G1 cells, implying arrested S-G2/M (and then G0/G1) progression rather than enhanced G1/S phase transition after Z-DON treatment. Further RNA sequencing of Z-DON treated erythroblasts and protein level examination to the expression changes of cell cycle-related factors will provide more insight to the pathways underlying erythroid function of TGM2. Together, our study suggested that TGM2 facilitates terminal erythroid differentiation, primarily at Baso stage. The function depends on its cross-linking activity, and involves dysregulated cell cycle progression.

## Data Availability

The datasets presented in this study can be found in online repositories. The names of the repository/repositories and accession number(s) can be found below: NCBI GEO under GSE229589.
