# Supplementary figures and images for: Transglutaminase 2 regulates terminal erythroid differentiation via cross-linking activity
Source: Front Cell Dev Biol. 2023 Apr 24;11:1183176. doi: 10.3389/fcell.2023.1183176 (PMC10164954; doi:10.3389/fcell.2023.1183176)

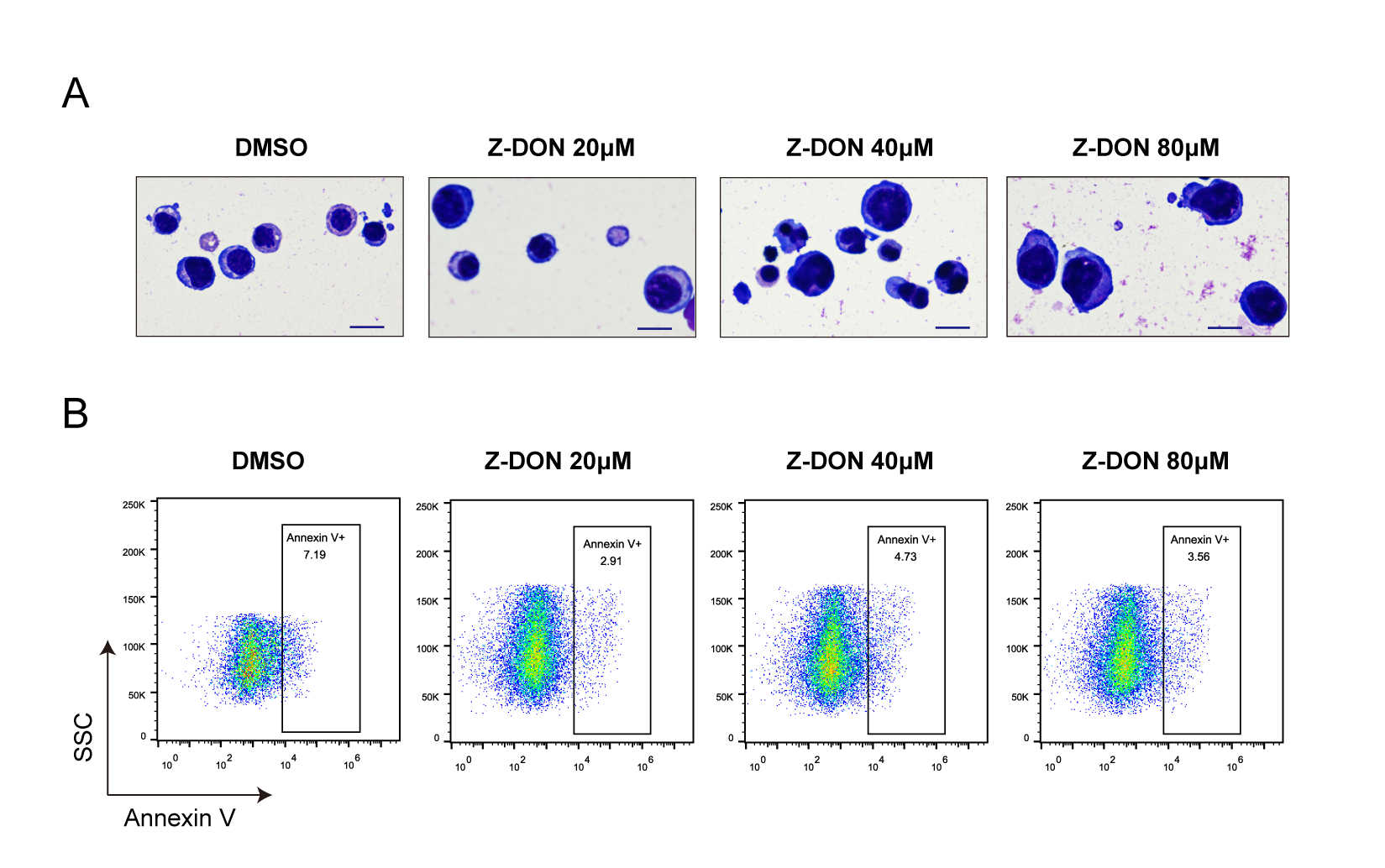

Supplement: Supplementary file 3 [file Image3.TIF]

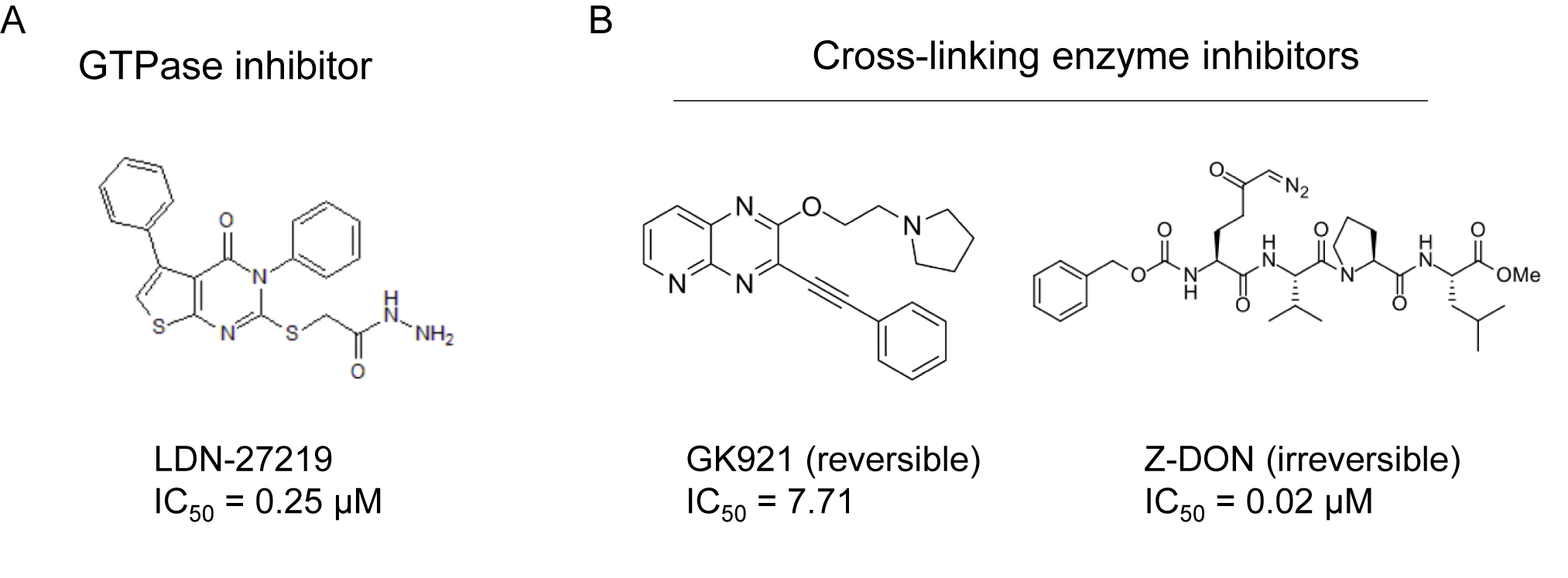

Supplement: Supplementary file 4 [file Image2.TIF]

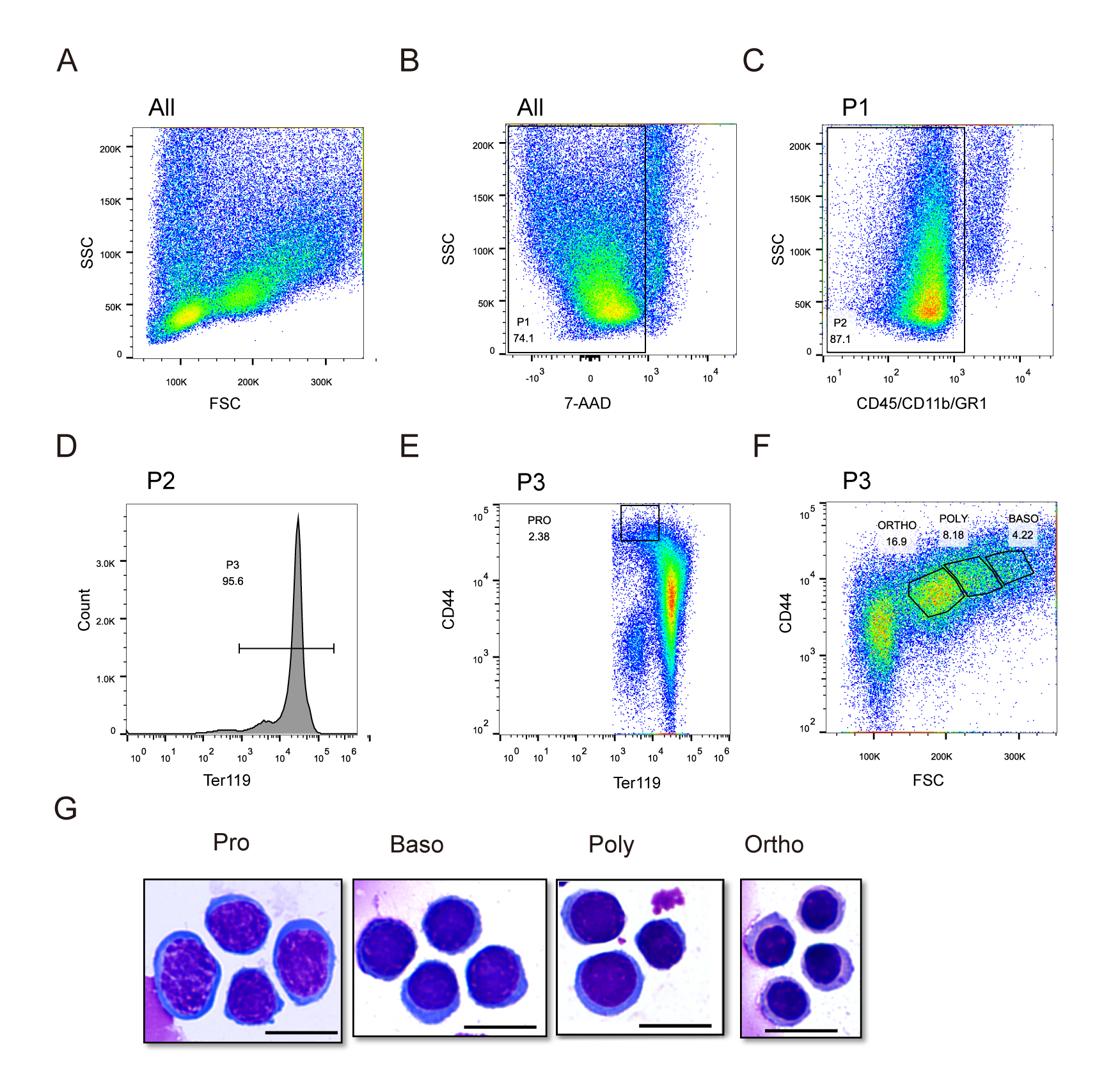

Supplement: Supplementary file 6 [file Image1.TIF]
