# Supplementary material for: Transglutaminase 2 regulates terminal erythroid differentiation via cross-linking activity
Source: Front Cell Dev Biol. 2023 Apr 24;11:1183176. doi: 10.3389/fcell.2023.1183176 (PMC10164954; doi:10.3389/fcell.2023.1183176)
Supplement: Supplementary file 7 [file DataSheet1.docx]

Supplementary Material

Transglutaminase 2 regulates terminal erythroid differentiation via cross-linking activity

Yingying Zhang^1^, Lifang Shi^1,2^, Ke Yang^1^, Xuehui Liu^1*^, Xiang Lv^1*^

^1^State Key Laboratory of Medical Molecular Biology, Haihe Laboratory of Cell Ecosystem, Department of Pathophysiology, Institute of Basic Medical Sciences, Chinese Academy of Medical Sciences & Peking Union Medical College, Beijing 100005, P.R. China.

^2^Changping Center for Disease Control and Prevention, Beijing 102200, P.R. China.

*** Correspondence:** Xuehui Liu, liuxuehui@ibms.pumc.edu.cn; Xiang Lv, lvxiang@pumc.edu.cn.

# Supplementary Figures and Tables

## Supplementary Figures


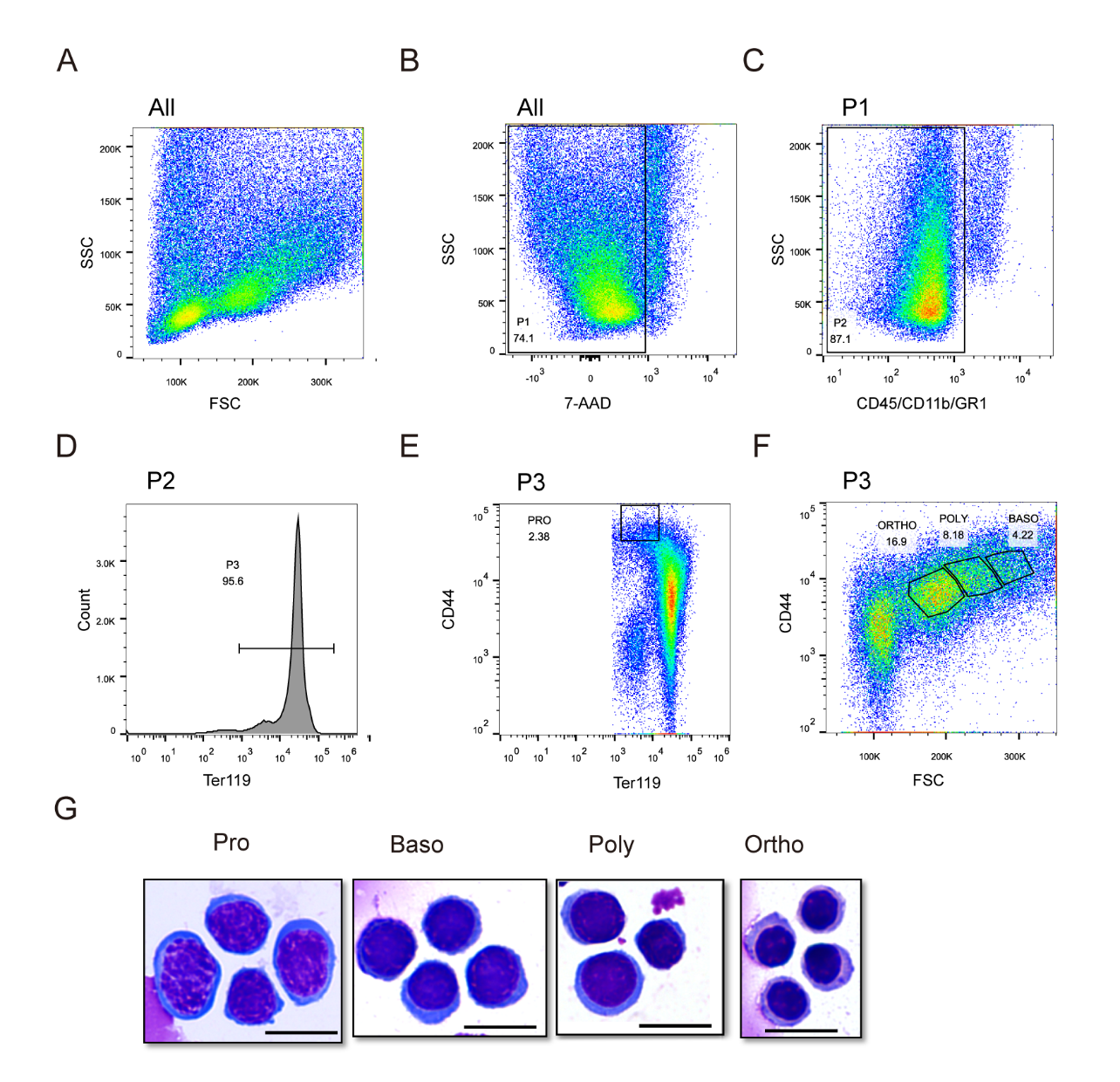


**Supplementary Figure S1.** **Flow cytometric analysis and isolation of mouse bone marrow erythroblasts of different maturation stages.** (**A**) Plot of FSC versus SSC of all the cells. (**B**) Plot of 7-AAD versus SSC of all the cells. The live cells (P1) were gated as 7-AAD negative cells. (**C**) Plot of CD45/CD11b/GR1 versus SSC within the P1 population. P2 were gated as CD45^-^CD11b^-^GR1^-^ cells (**D**) Histogram of Ter119 within the P2 population. P3 were gated as Ter119 positive cells. (**E**) Plot of CD44 versus Ter119 within the P3 cells. CD44^hi^Ter119^low^ cells were gated as P4 which is Pro cells. (**F**) Plot of CD44 versus FSC within P3 cells. P7, Baso cells. P6, Poly cells. P5, Ortho cells. (**G**) May-Grunwald Giemsa (MGG) stain of Pro, Baso, Poly and Ortho cells.


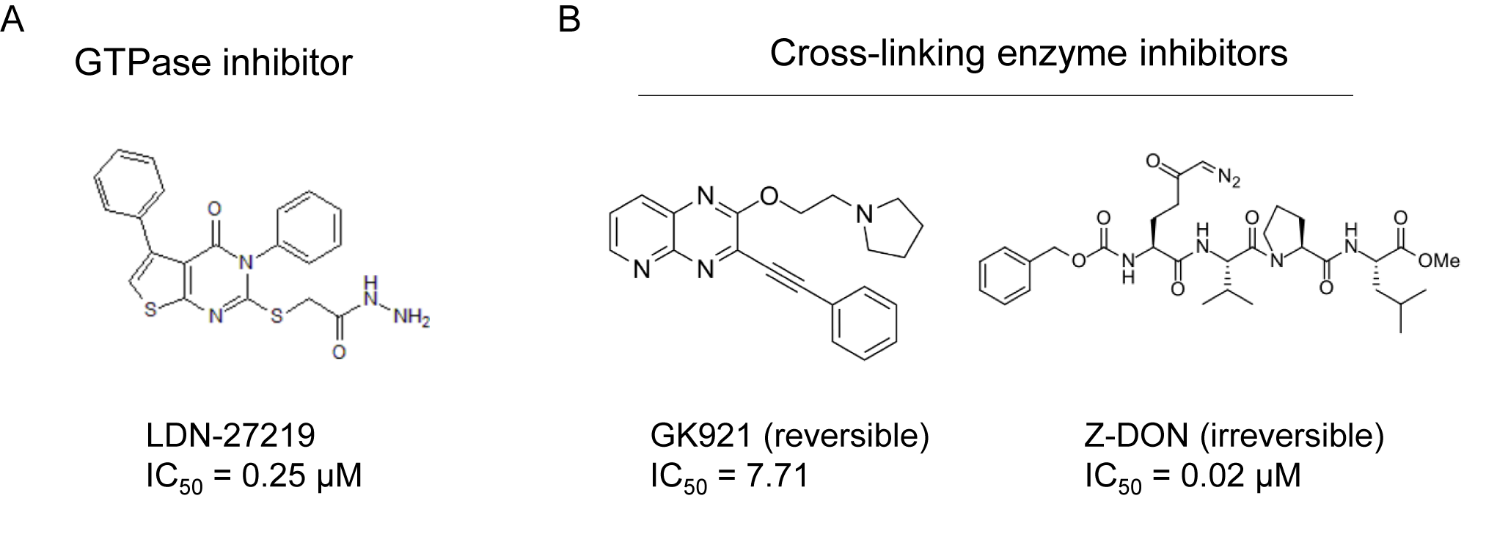


**Supplementary Figure S2.** **Structure formula of GTPase inhibitor and cross-linking enzyme inhibitors of TGM2 used in this study.**


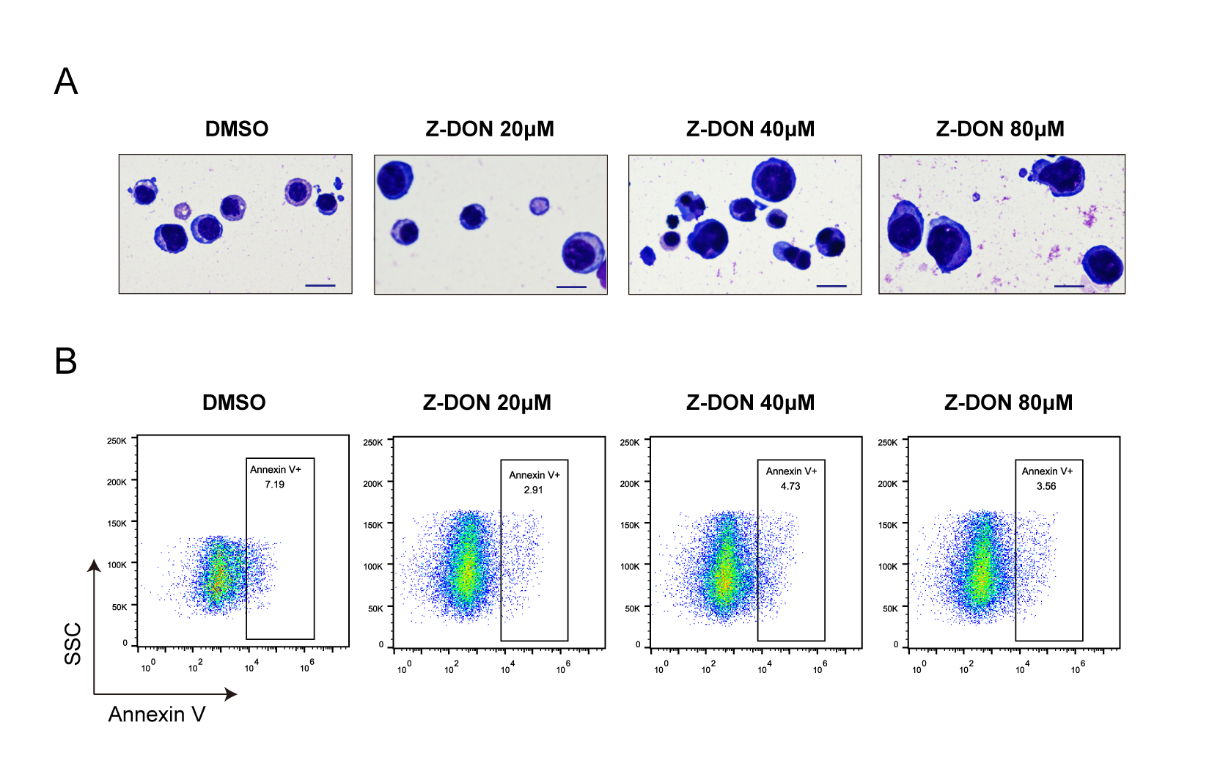


**Supplementary Figure S3. Z-DON arrests terminal erythroid differentiation of mouse fetal liver cells at the Baso stage and not induces cell apoptosis.** (A) May-Grunwald Giemsa (MGG) staining of cells differentiated for 4 days in the DMSO or in different concentrations of Z-DON. Photos were taken with a 63x objective of Zeiss brightfield microscope. Scale bar, 10 µm. (B) Flow cytometry analysis showing no effect of TGM2 inhibitor Z-DON on cell apoptosis of *in vitro* erythroid differentiated mouse fetal liver cells.

## Supplementary tables

**Supplementary Table S1. Real Time PCR primers.**

| **Genes** | **Primer sequences** | |
| --- | --- | --- |
|  | Forward (5’-3’） | Reverse (5’-3’） |
| Tgm2 | CGCAACAGGGCTTCATCTAC | CCCGACTACGGTTCTTCAGGA |
| Hbb | CCAGCCTCAGTGAGCTCCACT | GGCCCAGCACAATCACGAT |
| Ccne1 | GAAAAGCGAGGATAGCAGTCAG | CCCAATTCAAGACGGGAAGTG |
| Ccnd2 | TCCCGCAGTGTTCCTATTTC | CCAAGAAACGGTCCAGGTAA |
| Cdkn1a | CAGATCCACAGCGATATCCA | AGACAACGGCACACTTTGCT |
| Gadd45a | AGACCGAAAGGATGGACACG | GTACACGCCGACCGTAATG |
| Gata1 | CTTGGGATCACCCTGAACTC | AGGGCAGAATCCACAAACTG |
| Gata2 | CCTCCAGCTTCACCCCTAAG | ACAGGCATTGCACAGGTAGT |
| Klf1 | GCGGGAAGAGCTACACCAAG | GAGCGAACCTCCAGTCACAG |
| E2f4 | AGATCGCTGACAAGCTGATTG | CAAGGAGGGTATCTCCAGCA |
| E2f8 | ACCCTGCTGTGAATAACGACA | CTCTTCCCCGACACTCTTCAG |
| GAPDH | AGGTTGTCTCCTGCGACTTCA | CCAGGAAATGAGCTTGACAAA |

**Supplementary Table S2. Top 20 upstream regulators of terminal erythroid differentiation predicted between ortho and pro from mouse bone marrow via IPA analysis.**

| **Upstream Regulator** | **Molecule Type** | **Activation z-score** | **p-value of overlap** |
| --- | --- | --- | --- |
| TGM2 | enzyme | 6.132 | 3.79E-10 |
| IFNG | cytokine | 5.747 | 2.77E-16 |
| mir-223 | microRNA | 4.849 | 1.03E-20 |
| STAT1 | transcription regulator | 4.631 | 3.38E-10 |
| TNF | cytokine | 4.442 | 2.73E-18 |
| TLR9 | transmembrane receptor | 4.427 | 0.000000034 |
| IRF3 | transcription regulator | 4.412 | 1.39E-09 |
| TICAM1 | other | 4.011 | 0.00000333 |
| SPI1 | transcription regulator | 3.986 | 2.13E-10 |
| UCHL1 | peptidase | 3.9 | 7.81E-09 |
| HDAC2 | transcription regulator | 3.86 | 0.00000155 |
| TLR3 | transmembrane receptor | 3.843 | 0.000165 |
| SPIB | transcription regulator | 3.841 | 3.28E-10 |
| SMARCA4 | transcription regulator | 3.823 | 4.27E-13 |
| MYD88 | other | 3.675 | 0.000000544 |
| TLR7 | transmembrane receptor | 3.625 | 0.00245 |
| FOXO1 | transcription regulator | 3.535 | 0.000000299 |
| CD40LG | cytokine | 3.529 | 0.00105 |
| IL5 | cytokine | 3.508 | 0.000000735 |
| IFNAR1 | transmembrane receptor | 3.486 | 0.00000847 |

**Supplementary Table S3. The RNA expression (fpkm) of key erythroid transcriptional factors and cell cycle regulators (related to Fig. 4G and 5C) in mouse bone marrow erythroblasts of different maturation stages.**

| gene | pro_1 | pro_2 | pro_3 | baso_1 | baso_2 | baso_3 | poly_1 | poly_2 | poly_3 | ortho_1 | ortho_2 | ortho_3 |
| --- | --- | --- | --- | --- | --- | --- | --- | --- | --- | --- | --- | --- |
| Gata1 | 142.227 | 122.046 | 127.244 | 116.799 | 132.415 | 135.293 | 97.070 | 101.416 | 101.862 | 27.851 | 31.861 | 33.129 |
| Gata2 | 14.588 | 13.467 | 10.891 | 19.600 | 23.168 | 17.805 | 23.306 | 39.909 | 23.587 | 31.632 | 28.554 | 22.124 |
| Klf11 | 22.121 | 21.244 | 18.666 | 23.584 | 17.440 | 17.188 | 27.140 | 15.543 | 16.848 | 10.458 | 5.868 | 6.237 |
| E2f4 | 258.684 | 240.375 | 239.632 | 221.357 | 236.311 | 245.731 | 189.002 | 196.783 | 192.247 | 66.166 | 80.700 | 74.128 |
| E2f8 | 86.336 | 109.737 | 92.400 | 112.891 | 103.580 | 128.044 | 136.857 | 124.315 | 131.912 | 39.799 | 37.265 | 38.892 |
| Ccnd2 | 6.159 | 4.800 | 5.609 | 5.983 | 8.659 | 5.073 | 6.821 | 7.707 | 6.994 | 8.193 | 9.064 | 8.322 |
| Ccne1 | 138.137 | 129.882 | 125.791 | 56.422 | 69.497 | 73.765 | 56.415 | 52.447 | 61.084 | 14.483 | 15.484 | 16.257 |
| Cdkn1a | 4.923 | 6.646 | 6.960 | 11.608 | 7.821 | 5.610 | 13.499 | 9.915 | 5.467 | 15.268 | 10.828 | 7.332 |
| Gadd45a | 81.248 | 76.405 | 66.694 | 105.151 | 129.464 | 111.603 | 121.592 | 97.942 | 84.313 | 275.559 | 242.828 | 245.776 |
